# Folding and unfolding phylogenetic trees and networks

**DOI:** 10.1007/s00285-016-0993-5

**Published:** 2016-04-23

**Authors:** Katharina T. Huber, Vincent Moulton, Mike Steel, Taoyang Wu

**Affiliations:** 1 School of Computing Sciences, University of East Anglia, Norwich, NR4 7TJ UK; 2 School of Mathematics and Statistics, University of Canterbury, Christchurch, New Zealand

**Keywords:** Phylogenetic networks, Multi-labelled trees, Graph fibrations, Tree and network reconciliation, Universal cover of a digraph, 05C90, 92D15

## Abstract

Phylogenetic networks are rooted, labelled directed acyclic graphswhich are commonly used to represent reticulate evolution. There is a close relationship between phylogenetic networks and multi-labelled trees (MUL-trees). Indeed, any phylogenetic network *N* can be “unfolded” to obtain a MUL-tree *U*(*N*) and, conversely, a MUL-tree *T* can in certain circumstances be “folded” to obtain aphylogenetic network *F*(*T*) that exhibits *T*. In this paper, we study properties of the operations *U* and *F* in more detail. In particular, we introduce the class of stable networks, phylogenetic networks *N* for which *F*(*U*(*N*)) is isomorphic to *N*, characterise such networks, and show that they are related to the well-known class of tree-sibling networks. We also explore how the concept of displaying a tree in a network *N* can be related to displaying the tree in the MUL-tree *U*(*N*). To do this, we develop aphylogenetic analogue of graph fibrations. This allows us to view *U*(*N*) as the analogue of the universal cover of a digraph, and to establish a close connection between displaying trees in *U*(*N*) and reconciling phylogenetic trees with networks.

## Introduction

**Fig. 1:**
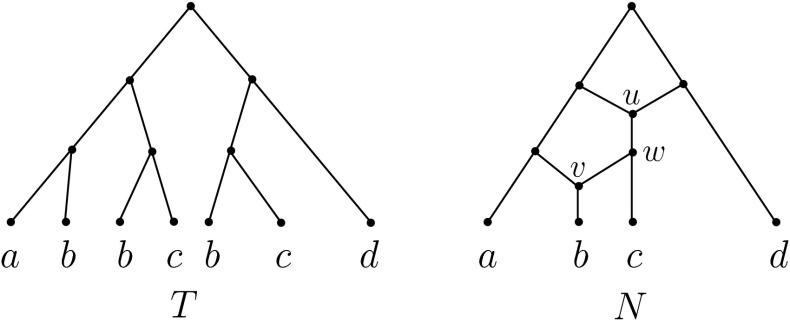
A MUL-tree *T* and its folding $N=F(T)$

Phylogenetic networks are rooted, directed acyclic graphs whose leaves are labelled by some set of species (see Sect. 2 for precise definitions of the concepts that we introduce in this section). Such networks are used by biologists to represent the evolution of species that have undergone reticulate events such as hybridization and there is much recent work on these structures (cf.e.g. Gusfield 2014; Huson et al. 2010). In Huber and Moulton (2006) a close relationship is described between phylogenetic networks and multi-labelled trees (MUL-trees), leaf-labelled trees where more than one leaf may have the same label. Essentially, it is shown that it is always possible to “unfold” a phylogenetic network *N* to obtain a MUL-tree *U*(*N*) and that, conversely, a MUL-tree *T* can under certain conditions be “folded” to obtain a phylogenetic network *F*(*T*) that exhibits *T*. We illustrate these operations in Fig. 1 (see Sect. 3 for more details). The tree *T* is a MUL-tree, and the network *N* is obtained by first inserting vertices into *T* and then folding up the resulting tree by identifying vertices in *T* to obtain *F*(*T*); the unfolding *U*(*N*) of *N* (which is essentially obtained by reversing this process) is precisely *T*.

Applications of the operations *F* and *U* include the construction of evolutionary histories of polyploids in terms of phylogenetic networks (Lott et al. 2009; Marcussen et al. 2015). In particular, polyploid organisms contain several copies of a genome, and if a tree is constructed from these genomes (or specific genes in these genomes) a MUL-tree can be obtained by labelling each leaf by the species that has the corresponding genome. By folding this MUL-tree a representation of the evolution of the species can then be obtained (in terms of a phylogenetic network), from the evolutionary history of the genomes. In this representation, vertices in the network with indegree two represent hybridisation events, where two parent species have produced a child which has the combined set of genomes of both of its parents.

In this paper, we study properties of the *F* and *U* operations in some detail and, in the process, show that they have some interesting connections with other areas such as gene tree/species network reconciliation (Wu and Zhang 2011; Zhang et al. 2011) and the theory of graph fibrations (Boldi and Vigna 2002). To do this, we begin by reviewing the concepts of MUL-trees and phylogenetic networks in the next section, and present some general properties of the folding and unfolding operations in Sect. 3. We then consider the interrelationship between the folding and unfolding operations.

More specifically, although it is always the case that *U*(*F*(*T*)) is isomorphic to *T* for any MUL-tree *T* (Huber and Moulton 2006), the same situation does not apply if the *U* and *F* operations are applied in the opposite order to some network as there are networks *N* for which *F*(*U*(*N*)) is not isomorphic to *N* (we give an example shortly in Fig. 3). Therefore, it is of interest to understand the networks *N* for which *F*(*U*(*N*)) is isomorphic to *N*. We call these *stable networks*. In Sect. 4, we present a characterization for stable phylogenetic networks (see Theorem 1). Using this result we are then able to show that the well-known class of binary, tree-sibling networks as defined in Cardona et al. (2009) are stable (see Corollary 1). We expect that stable networks could be of interest as they can provide a canonical representative for the set of all networks that display a particular MUL-tree (cf. Pardi and Scornavacca 2015 for choosing canonical representatives of networks that display a set of trees).

In Sect. 5, we show that the unfolding and folding operations are closely related to concepts that arise in the theory of graph fibrations (cf. Boldi and Vigna 2002 for a review of this area). In particular, we define the concept of a folding map between a MUL-tree and a phylogenetic network. As one consequence, we show that the unfolding of a network can be considered as a phylogenetic analogue of the universal cover of a digraph. This allows us to provide an alternative characterisation for stable networks (Corollary 3). It is worth noting that an alternative framework for considering maps between phylogenetic networks is developed in Willson (2012).

We then focus on the problem of displaying trees in networks. In Sect. 6, we demonstrate that it is NP-complete to decide whether or not a phylogenetic tree is displayed by a stable network (Theorem 5). This is of interest since in Kanj et al. (2008) it is shown that it is NP-complete to decide if a tree is displayed by a network, but in Iersel et al. (2010) it is shown that this problem is polynomial for certain special classes of networks (such as normal and tree-child networks).

Finally, in Sect. 7, we define and study a new way in which a tree may be displayed in a network: We say that a phylogenetic tree is *weakly displayed* by a phylogenetic network *N* if it is displayed by the MUL-tree *U*(*N*). Using the concepts developed in Sect. 5, we provide a characterization for when a tree is weakly displayed by a network in terms of a special type of tree reconciliation (Theorem 6). This characterisation allows us to show that, in contrast to displaying a tree, it is possible to decide in polynomial time whether or not a phylogenetic tree is weakly displayed by a phylogenetic network having the same leaf-set (Corollary 4).

## Definitions

Throughout this paper, we let *X* denote a finite set of size at least two. In addition, all graphs that we consider are connected.

### Rooted DAGs

Suppose *G* is a directed acyclic graph with vertex set *V*(*G*), arc set *A*(*G*) and a single root $\rho _G$. For an arc $a=(u,v)$ in *G*, we denote the *head* *v* of *a* by $h(a)=h_G(a)$, and its *tail* *u* by $t(a)=t_G(a)$. We say that two arcs *a* and $a'$ of *G* are *parallel* if $h(a)=h(a')$ and $t(a)=t(a')$ hold. If *v* is a vertex of *G*, then the *in-degree* of *v*, denoted by *indeg*(*v*), is the number of incoming arcs of *v*, and the *out-degree* of *v*, denoted by *outdeg*(*v*), is the number of outgoing arcs of *v*. We say that a vertex $v\in V(G)$ is *below* a vertex $w\in V(G)-\{v\}$ if there exists a directed path from *w* to *v* in *G*. We call a vertex *v* of *G* a *reticulation vertex* of *G*, if $outdeg(v)=1$ and $indeg(v)\ge 2$ holds. We call *v* a *tree vertex* of *G* if $indeg(v)=1$ and either $outdeg(v)=0$ holds, in which case we call *v* a *leaf* of *G*, or $outdeg(v)\ge 2$. We denote the set of leaves of *G* by *L*(*G*) and the set of *interior vertices* *v* of *G*, that is, *v* is neither the root nor a leaf of *G*, by $\mathring{V}(G)$.

**Fig. 2:**
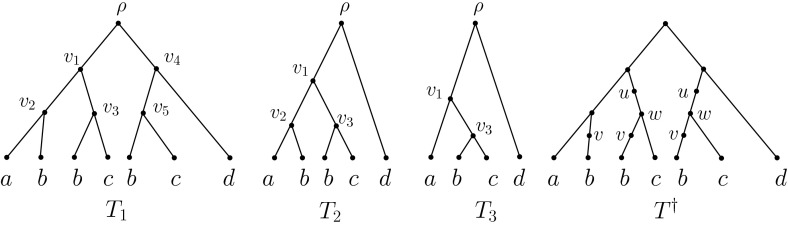
A sequence of MUL-trees $\tau : T_1,T_2,T_3$ and the intermediate pseudo MUL-tree $T^\dagger$ used by the folding operation *F* for the MUL-tree *T* in Fig. 1. Tree $T_2$ is obtained from $T_1$ by considering the maximal inextendible subMUL-tree $T_1(v_3)$ with root $v_3$. That is, we have $S_{v_3}=\{v_3,v_5\}$ and $T_2$ is constructed from $T_1$ by first deleting the subMUL-tree $T_1(v_5)$, then removing the arc $(v_4,v_5)$, and finally suppressing vertex $v_4$. Similarly, $T_3$ is obtained from $T_2$ by considering the maximal inextendible subMUL-tree on $\{b\}$ and incident with vertex $v_3$

### MUL-trees

We say that a multi-set *M* is *a multi-set on* *X* if the set resulting from *M* by ignoring the multiplicities of the elements in *M* is *X*. Following Huber and Moulton (2006), we define a *pseudo multi-labelled tree* ${\mathscr {T}}$ on *X*, or a *pseudo MUL-tree* on *X* for short, to be a pair $(T,\chi )$ consisting of a rooted directed tree *T* together with a labelling map $\chi : X\rightarrow {\mathscr {P}}(S)=2^S-\{\emptyset \}$ from *X* into the set ${\mathscr {P}}(S)$ of non-empty subsets of the leaf set $S=L(T)$ of *T* such that

(i) for all $x,y\in X$ distinct $\chi (x)\cap \chi (y)=\emptyset$, and
(ii) for every leaf $s\in S$ there exists some $x\in X$ with $s\in \chi (x)$.

In addition, we call $(T,\chi )$ a *MUL-tree on* *X* if the tree *T* does not have any vertices with in-degree one and out-degree one. If the map $\chi$ is clear from the context then we will write *T* rather than $(T,\chi )$ and if the set *X* is of no relevance to the discussion then we will call *T* a (pseudo) MUL-tree rather than a (pseudo) MUL-tree on *X*. For example, the tree *T* in Fig. 1 is a MUL-tree on $X=\{a,b,c,d\}$ and $T^\dagger$ in Fig. 2 is a pseudo MUL-tree on *X* that is not a MUL-tree on *X*. We say that two (pseudo) MUL-trees $(T_1, \chi _1)$ and $(T_2,\chi _2)$ on *X* are *isomorphic* if there is a digraph isomorphism $\xi :V(T_1)\rightarrow V(T_2)$ such that, for all $x\in X$ and $v\in V(T_1)$, we have $v\in \chi _1(x)$ if and only if $\xi (v)\in \chi _2(x)$.

Suppose *T* is a pseudo MUL-tree. For *v* a non-root vertex of *T*, we denote by *T*(*v*) the connected subgraph of *T* that contains *v* obtained by deleting the incoming arc of *v*. Clearly *T*(*v*) is a pseudo MUL-tree. We call a pseudo MUL-tree $T'$ a *pseudo subMUL-tree* of *T* if there exists a non-root vertex *v* of *T* such that *T*(*v*) and $T'$ are isomorphic. For *T* a MUL-tree we say that a subMUL-tree $T'$ of *T* is *inextendible* if there exist distinct vertices *v* and $v'$ of *T* such that $T'=T(v)$ and *T*(*v*) and $T(v')$ are isomorphic. Loosely speaking, a subMUL-tree of *T* is inextendible if *T* contains more than one copy of that subMUL-tree. We say that a subMUL-tree $T'$ of *T* is *maximal inextendible* if $T'$ is inextendible and any other inextendible subMUL-tree $T''$ of *T* that contains $T'$ as a subMUL-tree is isomorphic with $T'$. Note that although the definition of inextendible used in this paper is slightly different from the one in  (Huber and Moulton 2006), the maximal inextendible subMUL-trees coincide under both definitions.

To illustrate these definitions consider for example the MUL-tree *T* and its folding $N=F(T)$ depicted in Fig. 1. The three leaves labelled *b* are all inextendible subtrees of *T* and so are the two leaves labelled *c*. Each one of two subtrees of *T* of length two (ignoring the directions of the arcs of *T*) that have leaf label set $\{b,c\}$ is maximal inextendible.

### Phylogenetic networks

An *X* *-network* *N* is a rooted directed acyclic graph, in which parallel arcs are allowed,

such that

(i) there exists a unique root $\rho _N$ of *N* that has in-degree zero and out-degree at least two,
(ii) every vertex of *N* except the root is either a reticulation vertex or a tree vertex,
(iii) there exists no vertex of in-degree one and out-degree one, and
(iv) the set *L*(*N*) of leaves of *N* is *X*.

A *phylogenetic network* ( *on* *X*) is an *X*-network that does not have parallel arcs. A *phylogenetic tree on* *X* is a phylogenetic network on *X* that has no reticulation vertices. We say that a phylogenetic network *N* is *binary* if the degree of every reticulation vertex and every non-leaf tree-vertex is three and $outdeg(\rho _N)=2$. Finally, we say that two *X*-networks *N* and $N'$ are *isomorphic* if there exists a bijection $\kappa :V(N)\rightarrow V(N')$ such that for all vertices $u,v\in V(N)$ the number of arcs in *N* with head *u* and tail *v* equals the number of arcs in $N'$ with head $\kappa (u)$ and tail $\kappa (v)$, and $\kappa$ is the identity on *X*.

## Folding and unfolding

In this section, we recall the unfolding and folding operations mentioned in the introduction that were first proposed in Huber and Moulton (2006) (see also Huber et al. 2012 for the binary case).

We first describe the unfolding operation *U* which constructs a pseudo MUL-tree $U^*(N)$ from an *X*-network *N* as follows:

- the vertices of $U^*(N)$ are the directed paths in *N* that start at $\rho _N$,
- there is an arc from vertex $\pi$ in $U^*(N)$ to vertex $\pi '$ in $U^*(N)$ if and only if $\pi ' = \pi a$ holds for some arc *a* in *N* (i.e. $\pi '$ is the path in *N* that extends the path $\pi$ in *N* by the arc *a*), and
- the vertices in $U^*(N)$ that start at $\rho _N$ and end at some *x* in *X* are labelled by *x*.

The MUL-tree obtained by suppressing all in-degree one and out-degree one vertices in $U^*(N)$, if there are any, is denoted by *U*(*N*). In Huber and Moulton (2006) it is shown that *U*(*N*) is indeed a MUL-tree.

We denote for all vertices *v* of an directed graph *G* as in Sect. 2.1 the set of children of *v* by *ch*(*v*) and say that an *X*-network *N* *exhibits* a MUL-tree *T* if the MUL-trees *U*(*N*) and *T* are isomorphic. In particular, any *X*-network *N* exhibits the MUL-tree *U*(*N*). Note that there exist MUL-trees *T* for which there is no phylogenetic network that exhibits *T* (for example, the binary MUL-tree with two leaves both labelled by the same element).

We now describe the folding operation *F* for constructing an *X*-network *F*(*T*) from a MUL-tree *T* introduced in (Huber and Moulton 2006, p. 628). This operation can be thought of intuitively as the reverse of the unfolding operation *U*, and it works by repeatedly finding a maximal inextendible subMULtree, subdividing the incoming arcs of the roots of the subMUL-trees that are isomorphic with it, and finally identifying the subdivision vertices and removing all but one copy of that subMUL-tree. This continues until an *X*-network is obtained or, equivalently, no further maximal inextendible subMUL-tree can be found. Formally, a pseudo MUL-tree $T^\dagger$ is constructed from *T* to guide this process. To do this we need to define a sequence $\tau : T=T_1, T_2,...$ of MUL-trees. Suppose $i\ge 1$ is such that we have already constructed tree $T_i$. Then we obtain $T_{i+1}$ as follows: If there is no inextendible subMUL-tree of $T_i$, we declare $T_i$ to be the last tree in $\tau$. Otherwise, we take a maximal inextendible subMUL-tree of $T_i$. Let *v* be the root of this tree and let $S_v$ be the subset of vertices *w* of $T_i$ with $T_i(w)$ isomorphic to $T_i(v)$. Then, to obtain $T_{i+1}$, for each $w \in S_v-\{v\}$ we remove the subtree $T_i(w)$ and the arc with head *w* from $T_i$. If this has rendered the root $\rho _{T_i}$ of $T_i$ a vertex with out-degree one then we collapse the remaining arc with tail $\rho _{T_i}$. Otherwise, we suppress the resulting vertex with in-degree and out-degree one. For the MUL-tree *T* in Fig. 1, an example of such a MUL-tree sequence is depicted in Fig. 2.

Now, to obtain $T^\dagger$, we consider each tree in $\tau$ other than *T* in turn. Let $i\ge 2$ and assume that $v\in V(T)$ is such that $T_i$ is constructed from $T_{i-1}$. Then we subdivide all of those arcs *a* in *T* for which $T(h_T(a))$ and *T*(*v*) are isomorphic (as pseudo MUL-trees). The pseudo MUL-tree obtained once the last element in $\tau$ has been processed is $T^\dagger$. Note that although the pseudo MUL-tree sequences are not necessary unique, they all result in the same intermediate tree $T^\dagger$.

Finally, to obtain *F*(*T*), we define an equivalence relation $\sim _{T^\dagger }$ on $V(T^\dagger )$ that identifies all pairs of vertices *v*, *w* in $V(T^\dagger )$ with $T^\dagger (v)$ isomorphic with $T^\dagger (w)$ (as pseudo-MUL-trees), and let *F*(*T*) be the *X*-network obtained by taking the quotient of $T^\dagger$ by $\sim _{T^\dagger }$. More precisely, let *G*(*T*) denote the DAG with vertex set $\{[u] _{\sim _{T^\dagger }}\,:\, u\in V(T^\dagger ) \}$ and (multi)-set of arcs obtained by joining any two vertices $u,v\in V(T^\dagger )$ for which $[u]_{\sim _{T^\dagger }}\not =[v]_{\sim _{T^\dagger }}$ holds by $m\ge 0$ arcs $([u]_{\sim _{T^\dagger }},[v]_{\sim _{T^\dagger }})$ if and only if for one (and hence for all) $u'\in [u]$ the size of $ch(u')\cap [v]_{\sim _{T^\dagger }}$ is *m*. The *X*-network obtained from *G*(*T*) by suppressing all vertices of indegree one and outdegree one and defining the leaf labels in the natural way is *F*(*T*).

For example, consider the MUL-tree *T* in Fig. 1. Then the pseudo MUL-tree $T^\dagger$ is depicted in Fig. 2. The two vertices labelled *u* in $T^\dagger$ make up $[u]_{\sim _{T^\dagger }}$ and the vertex in *F*(*T*), representing $[u]_{\sim _{T^\dagger }}$ is labelled *u*. Similarly, the two vertices labelled *w* in $T^\dagger$ make up $[w]_{\sim _{T^\dagger }}$ which we again represent in *F*(*T*) in terms of *w*. Clearly $[u]_{\sim _{T^\dagger }}\not =[w]_{\sim _{T^\dagger }}$ and $|ch(u')\cap [w]_{\sim _{T^\dagger }}|=1$ holds for all $u'\in [u]_{\sim _{T^\dagger }}$. Hence, there is precisely one arc in *F*(*T*) from *u* to *w*.

Note that any MUL-tree *T* is isomorphic with *U*(*F*(*T*)) (as MUL-trees) (Huber and Moulton 2006). Thus, if there is no risk of confusion we will sometimes identify *T* and *U*(*F*(*T*)). Also, note that if *T* is binary, then *F*(*T*) is *semi-resolved*, that is, every tree vertex in *F*(*T*) has out-degree 2. Moreover, in Huber and Moulton (2006), Proposition 3 it is shown that if *F*(*T*) is semi-resolved, then *F*(*T*) has the minimum number of reticulation vertices amongst all phylogenetic networks that exhibit *T*.

**Fig. 3:**
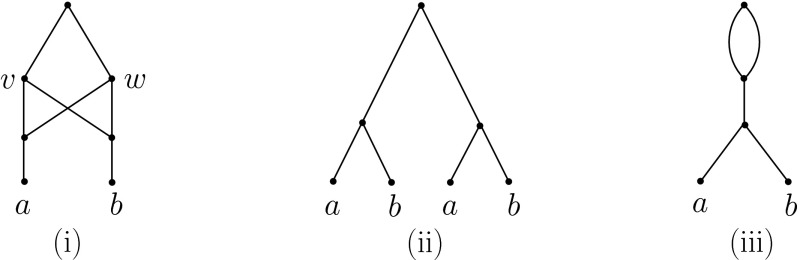
**(i)** A phylogenetic network *N*, **(ii)** *U*(*N*), and **(iii)** the *X*-network *F*(*U*(*N*)). Clearly, *N* and *F*(*U*(*N*)) are not isomorphic

In general, the folding of an arbitrary MUL-tree on *X* need not be a phylogenetic network. For example, consider the MUL-tree *U*(*N*) in Fig. 3(ii); then its fold up *F*(*U*(*N*)) is not a phylogenetic network as it contains parallel arcs. We now characterize those MUL-trees *T* for which *F*(*T*) *is* a phylogenetic network.

### **Proposition 1**

Suppose *T* is a binary MUL-tree on *X*. Then *F*(*T*) is a phylogenetic network if and only if there is no pair of distinct vertices *v*, *w* in *T* which share a parent in *T* and are such that *T*(*v*) and *T*(*w*) are isomorphic.

### *Proof*

We prove the claim that if *F*(*T*) is a phylogenetic network then there is no pair of distinct vertices in *T* with the stated property by establishing the contrapositive. Suppose *T* is a binary MUL-tree on *X* that contains two distinct vertices *v* and *w* which share a parent in *T* and are such that *T*(*v*) and *T*(*w*) are isomorphic. Without loss of generality, we may assume that *v* and *w* are such that there exist no two vertices $v'\not = v$ and $w'\not =w$ of *T* on the directed paths from the root of *T* to *v* and *w*, respectively, such that $T(v')$ and $T(w')$ are isomorphic and the parent of $v'$ is also the parent of $w'$. Thus, *T*(*v*) is inextendible. If *T*(*v*) is maximal inextendible then *F*(*T*) contains a parallel arc and so *F*(*T*) is not a phylogenetic network, as required. So, assume that *T*(*v*) is not maximal inextendible. Then there must exist some vertex $v''$ in *T* distinct from *v* and *w* such that $T(v'')$ is maximal inextendible and *T*(*v*) is a subMUL-tree of $T(v'')$.

Let $z_0 = v'', z_1, \dots , z_l$, $l \ge 0$, denote the vertices on the directed path from $v''$ to *v* such that $T(z_i)$ is inextendible and is rendered maximal inextendible during the folding of *T*. Then $z_l = v$ must hold as every MUL-tree $T(z_i)$, $0 \le i <l$, must contain both *T*(*v*) and *T*(*w*) as a subMUL-tree. Thus, *T*(*v*) is rendered maximal inextendible at some stage in the construction of *F*(*T*). Applying the operation *F* to *T*(*v*) introduces a parallel arc into *F*(*T*) and thus *F*(*T*) is not a phylogenetic network, as required.

Conversely, suppose that *T* is a binary MUL-tree on *X* such that there exist no two vertices *v*, *w* in *T* which share a parent in *T* such that *T*(*v*) and *T*(*w*) are inextendible. Assume for contradiction that *F*(*T*) is not a phylogenetic network. Then *F*(*T*) must contain parallel arcs *a* and $a'$. Put $v=t(a)=t(a')$ and $w=h(a)=h(a')$. Then *v* is a tree vertex and *w* is a reticulation vertex of *F*(*T*). Let *z* denote the unique child of *w* in *F*(*T*). Note that since the folding operation implies that *F*(*T*) cannot contain an arc both of whose end vertices are reticulation vertices, *z* must in fact be a tree vertex in *F*(*T*).

Now, let $\gamma , \gamma '$ denote two directed paths from the root $\rho _{F(T)}$ of *F*(*T*) to *z* which contain arcs *a* and $a'$, respectively, and which differ only on those arcs. Denoting the MUL-tree *U*(*F*(*T*)) on *X* by $T^*$, the subMUL-trees $T^*(\gamma )$ and $T^*(\gamma ')$ of $T^*$ are isomorphic. But this is impossible, since there is a directed path $\gamma ''$ from $\rho _{F(T)}$ to *v* such that $\gamma ''$ is the parent of both $\gamma$ and $\gamma '$ in $T^*$ which is isomorphic to *T*. Thus, *F*(*T*) must be a phylogenetic network. $\square$

As mentioned above, the folding operation *F* can be considered as the reverse of the operation *U*. However, there exist phylogenetic networks *N* such that *F*(*U*(*N*)) is not isomorphic to *N* (see e.g. Fig. 3). Therefore, it is of interest to understand those networks *N* for which *F*(*U*(*N*)) and *N* are isomorphic.

## Stable networks

In this section, we shall give a characterization of phylogenetic networks *N* for which *F*(*U*(*N*)) is isomorphic to *N*. We call such networks *stable*.

We start by recalling the definition of an irreducible network (Huber and Moulton 2006). Suppose that *N* is a phylogenetic network on *X*. We call two distinct tree vertices *v* and *w* in *N* *identifiable* if there exist directed paths $\gamma _v$ from the root $\rho _N$ of *N* to *v* and $\gamma _w$ from $\rho _N$ to *w* such that the subMUL-trees $T(\gamma _v)$ and $T(\gamma _{w})$ of *U*(*N*) are isomorphic. In addition, we say that *N* is *irreducible* if it does not contain an identifiable pair of tree vertices. To illustrate, the network *N* depicted in Fig. 3(i) is not irreducible, since the two vertices *v* and *w* are identifiable.

If *N* is a phylogenetic network then let *Ret*(*N*) denote the set of reticulation vertices of *N*. We call *N* *compressed* if the child of each vertex in *Ret*(*N*) is a tree vertex. Note that in Cardona et al. (2009), this property is taken as part of the definition of a phylogenetic network, the rationale being that we cannot expect to reconstruct the order in which hybridization events occur.

### **Theorem 1**

Suppose that *N* is a semi-resolved phylogenetic network. Then the following statements are equivalent.

(i) *N* is stable.
(ii) *N* is compressed and irreducible.
(iii) *N* is compressed and there does not exist a pair of distinct tree vertices *v*, *w* in *N* such that $ch(v)=ch(w)$.

### *Proof*

(ii) $\Rightarrow$ (iii): Suppose that (ii) holds and assume for contradiction that there exists a pair of distinct tree vertices *v*, *w* in *N* such that $ch(v)=ch(w)$. Then $ch(v)\subseteq Ret(N)$. Since *N* is semi-resolved we have $|ch(v)|=2$. Let $\{a,b\}=ch(v)$. Since *N* is compressed the children $a'$ and $b'$ of *a* and *b*, respectively, are tree-vertices of *N*. Let $\gamma _{a'}^v$ and $\gamma _{a'}^w$ denote the directed paths from the root $\rho _N$ of *N* to $a'$ that cross *v* and *w*, respectively. Similarly, let $\gamma _{b'}^v$ and $\gamma _{b'}^w$ denote the directed paths in *N* from $\rho _N$ to $b'$ that cross *v* and *w*, respectively. Then the subMUL-trees $T(\gamma _{a'}^v)$ and $T(\gamma _{a'}^w)$ of *U*(*N*) are isomorphic and so are the subMUL-trees $T(\gamma _{b'}^v)$ and $T(\gamma _{b'}^w)$. Let $\nu$ denote the subpath obtained from $\gamma _{a'}^v$ by terminating at *v*. Similarly, let $\mu$ denote the subpath obtained from $\gamma _{a'}^w$ by terminating at *w*. Then the MUL-tree obtained from $T(\gamma _{a'}^v)$ and $T(\gamma _{b'}^v)$ by adding the vertex labelled $\nu$ and the arcs $(\nu ,\gamma _{a'}^v)$ and $(\nu ,\gamma _{b'}^v)$ is $T(\nu )$. Similarly, the MUL-tree obtained from $T(\gamma _{a'}^w)$ and $T(\gamma _{b'}^w)$ by adding the vertex labelled $\mu$ and the arcs $(\mu ,\gamma _{a'}^w)$ and $(\mu ,\gamma _{b'}^w)$ is $T(\mu )$. Since $T(\nu )$ and $T(\mu )$ are clearly isomorphic it follows that *v*, *w* is an identifiable pair in *N*. Hence *N* is not irreducible which provides the required contradiction.

(iii) $\Rightarrow$ (ii): Suppose that (iii) holds and assume for contradiction that *N* is not irreducible. Then *N* contains an identifiable pair of vertices *v*, *w*. Without loss of generality, we may assume that *v* and *w* are such that there are no vertices $v'$ and $w'$ below *v* and *w*, respectively, that also form an identifiable pair.

To obtain the required contradiction, we first claim that *ch*(*v*) and *ch*(*w*) are contained in *Ret*(*N*). Suppose that $s \in ch(v)$. For all non-root vertices *u* of *N* let $\gamma _u$ denote a directed path from the root $\rho _N$ of *N* to *u*. If *s* is a leaf of *N* then, since *v* and *w* are an identifiable pair, the MUL-trees $T(\gamma _v)$ and $T(\gamma _w)$ are isomorphic and the underlying bijection is the identity on *X*. Hence, $s\in ch(w)$ holds too and, so, $s\in Ret(N)$ which is impossible as *s* is a leaf of *N*.

If *s* is a non-leaf tree-vertex of *N* then, since $T(\gamma _v)$ and $T(\gamma _w)$ are isomorphic and every tree vertex *z* of *N* gives rise to a subset of vertices in the MUL-tree *U*(*N*), it follows that there exists a non-leaf tree vertex $s'$ below *w* such that $T(\gamma _s)$ and $T(\gamma _{s'})$ are isomorphic. By the choice of *v* and *w*, we cannot have that *s* and $s'$ form an identifiable pair and so $s=s'$ must hold. Hence, $s\in Ret(N)$, which is impossible as *s* is assumed to be a tree vertex of *N*. Since every non-root vertex of *N* is either a tree-vertex or a reticulation vertex of *N*, it follows that $ch(v)\subseteq Ret(N)$. Similar arguments imply that $ch(w)\subseteq Ret(N)$ also holds which completes the proof of the claim.

To complete the proof, assume for contradiction that there exists some $s\in ch(v)-ch(w)$. Then, $s\in Ret(N)$, by the previous claim. Since *N* is compressed, the child $s'$ of *s* must be a tree-vertex of *N*. Since $T(\gamma _v)$ and $T(\gamma _w)$ are isomorphic it follows that there exists a tree vertex *r* in *N* below *w* such that $T(\gamma _{s'})$ and $T(\gamma _r)$ are isomorphic. Note that $s'\not =r$ as otherwise $s'$ must be a reticulation vertex of *N* which is impossible. Hence, $s'$ and *r* form an identifiable pair in *N* with $s'$ below *v* and *r* below *w* which is impossible in view of the choice of *v* and *w*. Thus, $ch(v)\subseteq ch(w)$. Similar arguments imply that $ch(w)\subseteq ch(v)$ and so $ch(w)= ch(v)$ must hold, as required. But this is impossible in view of (iii).

(i) $\Rightarrow$ (ii): This follows by Huber and Moulton (2006), Theorem 3.

(ii) $\Rightarrow$ (i): Suppose that *N* is compressed and irreducible. Let $N^b$ and $F(U(N))^b$ denote some binary resolution of *N* and *F*(*U*(*N*)), respectively. Since *N* is irreducible so is $N^b$, and since *N* exhibits *U*(*N*) so does $N^b$. Hence, by applying Huber and Moulton (2006), Corollary 2 to $N^b$ and $F(U(N))^b$ and using the assumption that *N* is compressed, it follows that *N* is stable. $\square$

As an immediate corollary (Corollary 1) of this last theorem, we see that the collection of binary, stable phylogenetic networks contains a well-known class of phylogenetic networks. More specifically, suppose that *N* is a phylogenetic network. A vertex *w* of *N* distinct from some vertex *v* of *N* is a *sibling* of *v* if *v* and *w* share the same parent, and a sibling that is a tree vertex is called a *tree-sibling* vertex. In addition, *N* is called a *tree-child* network if every non-leaf vertex of *N* has a child that is a tree vertex of *N* (Cardona et al. 2008), and *N* is called a *tree-sibling* network if every reticulation vertex of *N* has a tree-sibling (Cardona et al. 2009). Note that a tree-child network is a tree-sibling network.

### **Corollary 1**

Suppose *N* is a binary, compressed, tree-sibling network. Then *N* is stable.

**Fig. 4:**
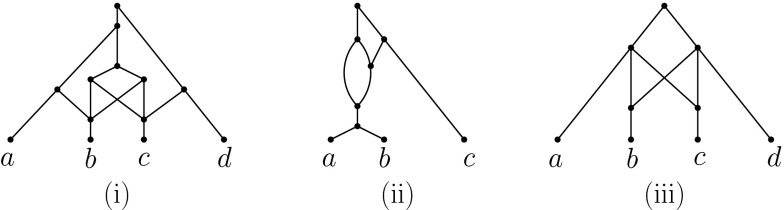
**(i)** The network on $X=\{a,b,c,d\}$ is semi-resolved, compressed tree-sibling but not stable. **(ii)** The network on $X=\{a,b,c\}$ is binary, stable but not tree-sibling. **(iii)** The network on $X=\{a,b,c,d\}$ is non-binary, tree-child but not stable

By Corollary 1, it follows that the fold up of an unfolded binary, compressed, tree-sibling network is the network itself and that it cannot contain a pair of distinct tree vertices that have the same set of children. Note that there exist semi-resolved, compressed, tree-sibling networks that are not stable (Fig. 4(i)), binary, stable phylogenetic networks that are not tree-sibling (Fig. 4(ii)), and non-binary, tree-child networks that are not stable (Fig. 4(iii)).

## Folding maps

In this section, we explore a relationship between the folding/unfolding operations and graph fibrations. For simplicity, we shall follow the presentation of the latter topic in Boldi and Vigna (2002). Results from this section will be used to establish a main result in Sect. 7.

Recall that the head of an arc *a* in an *X*-network *N* is denoted by $h_N(a)$ and its tail by $t_N(a)$. Now, suppose that $(T,\chi )$ is a pseudo MUL-tree on *X* and that *N* is a phylogenetic network on *X*. An *X* *-morphism* $f:T \rightarrow N$ is a pair of functions $f_V:V(T) \rightarrow V(N)$, $f_A:A(T) \rightarrow A(N)$ such that (i) for all $a \in A(T)$, we have $h_N(f_A(a))=f_V(h_T(a))$ and $t_N(f_A(a))=f_V(t_T(a))$, and (ii) if $v \in L(T)$ with $v \in \chi (x)$, $x \in X$, then $f_V(v)=x$. An *X*-morphism *f* is called a *rooted* *X* *-morphism* if $f_V(\rho _T)=\rho _N$ also holds. In case the context is clear, we denote both $f_V$ and $f_A$ by *f*. We call an *X*-morphism $f:T \rightarrow N$ a *folding map* 1 if both maps $f_V, f_A$ are surjective, and for each arc $a \in A(N)$ and $v \in V(T)$ such that $f(v)=t(a)$ there is a unique arc $\widetilde{a^v} \in A(T)$ (the *lifting of the arc* *a* *at* *v*) such that $f(\widetilde{a^v})=a$ and $t(\widetilde{a^v})=v$. Note that a folding map is necessarily a rooted *X*-morphism. We call the inverse image $f^{-1}(v)$, $v \in V(N)$, the *fibre* over *v*. Informally, the fibre over *v* is the subset of *V*(*T*) that is mapped to *v* under *f*. For example, for the tree $T^{\dagger }$ and the phylogenetic network *N* depicted in Fig. 2 and Fig. 1, respectively, the fibers of the vertices *u*, *v* and *w* in *N* are given by the vertices of $T^{\dagger }$ labelled with the same letters.

We begin by stating a result which illustrates how folding maps naturally arise from the unfolding *U*(*N*) of a network *N*. This result is an analogue of Boldi and Vigna (2002), Theorem 15; the proof is quite similar and straight-forward and so we omit it.

### **Theorem 2**

Let *N* be a phylogenetic network on *X*. Then the map $f^*:U^*(N) \rightarrow N$ that takes each vertex $\pi$ in $U^*(N)$ to its last vertex, and each arc of $U^*(N)$ to the corresponding arc in *N* is a folding map.

As we shall now show, the folding *F*(*T*) of a MUL-tree *T* can also give rise to a folding map. In analogy with Boldi and Vigna (2002), p. 25, we say that an equivalence relation $\sim$ on the vertex set *V*(*T*) of a pseudo MUL-tree *T* satisfies the *local out-isomorphism property* (*LOIP*) if the following holds for all $v,w \in V(T)$.

- *LOIP:* If $v \sim w$ then there is a bijection $\xi$ from the set of arcs in *T* with tail *v* to the set of arcs in *T* with tail *w* such that $h(a) \sim h(\xi (a))$, for all arcs *a* in *T* with tail *v*.

We now use the LOIP-property to characterize when equivalence relations on MUL-trees give rise to folding maps (cf. Boldi and Vigna 2002, Theorem 2). To aid clarity of presentation, we denote the parent of a non-root vertex *v* in a rooted directed tree by *pa*(*v*).

### **Theorem 3**

Suppose that $(T,\chi )$ is a pseudo MUL-tree on *X*, and $\sim$ is an equivalence relation on *V*(*T*). Then the equivalence classes $[.]_\sim$ are the fibres of a folding map $f:T \rightarrow N$ (for some phylogenetic network *N* on *X*) if and only if $\sim$ satisfies the following five properties:

(i) LOIP,
(ii) for all $v \in V(T)$ with in-degree and out-degree 1, $|[v]_{\sim }| \ge 2$, and $pa(v) \not \sim pa(v'')$ for some $v'' \in [v]_{\sim }$,
(iii) for all $v\in V(T)$ with in-degree 1 and out-degree not equal to 1, $pa(v) \sim pa(v')$ for all $v' \in [v]_{\sim }$,
(iv) for all $x \in X$ and $v \in \chi (x)$, $[v]_{\sim } = \chi (x)$, and
(v) for all $v\in V(T) - \{\rho _T\}$, $pa(v) \not = pa(v')$ for all $v' \in [v]_\sim$ distinct from *v*.

### *Proof*

Assume first that *N* is a phylogenetic network on *X* and $f:T\rightarrow N$ is a folding map such that the equivalence classes $[.]_\sim$ of $\sim$ are the fibres of *f*. For each $v,w \in V(T)$ with $v \sim w$, define a map $\xi$ from the set of arcs *a* in *T* with tail *v* to the set of arcs in *T* with tail *w* by putting $\xi (a)$ equal to $\widetilde{f(a)^w}$. Then $f(h(\xi (a)))=f(h(\widetilde{f(a)^w})) =h(f(\widetilde{f(a)^w}))=h(f(a))=f(h(a))$. Hence $h(\xi (a)) \sim h(a)$, and so $\sim$ satisfies (i). Moreover, as *N* is a phylogenetic network, it is straight-forward to check that (ii) must hold as no vertex in *N* can have in-degree and out-degree 1, (iii) must hold as every vertex of *N* that is not the root of *N* is either a reticulation vertex or a tree vertex (but not both), and that (iv) must hold as all elements in $\chi (x)$ must be mapped by *f* to a vertex labeled by *x* which has in-degree 1. Finally, (v) follows from the fact that *N* does not contain parallel arcs.

Conversely, assume that $\sim$ is an equivalence relations on *V*(*T*) that satisfies properties (i)–(v). To simplify notation, put $[u]=[u]_\sim$ for all vertices *u* in *V*(*T*). Let $T/\sim$ be the network obtained by taking the quotient of *T* by $\sim$ (as described in Sect. 3). In particular, $T/\sim$ is a rooted DAG with vertex set $V(T)/\sim$, and ([*u*], [*v*]) an arc in $T/\sim$ for $u,v \in V(T)$ if and only if $(u',v') \in A(T)$ for some $u'\in [u]$ and $v'\in [v]$ (note that this definition is independent of the choice of $u'$ and $v'$). In addition, we identify each leaf [*u*] in $T/\sim$ with the necessarily unique element *x* in *X* with $[u]=\chi (x)$ whose existence follows from property (iv). It is straight-forward to check that properties (i)–(v) ensure that $T/\sim$ is a phylogenetic network on *X*.

Now, define $f:T \rightarrow T/\sim$ to be the *X*-morphism that maps each vertex *u* in *V*(*T*) to its equivalence class [*u*], and each arc (*u*, *v*) in *A*(*T*) to the arc ([*u*], [*v*]). It is straight-forward to check that *f* is a folding map as properties (i) and (iv) imply that *f* yields a well-defined surjective *X*-morphism from *T* to $T/\sim$ that satisfies the aforementioned arc lifting property. $\square$

Given a MUL-tree *T*, consider the equivalence relation $\sim _{T^\dagger }$ on the vertex set $V(T^\dagger )$ of the pseudo MUL-tree $T^\dagger$ defined in Sect. 3. Since $\sim _{T^\dagger }$ satisfies properties (i)–(v) of the last theorem it follows that, in case *F*(*T*) is a phylogenetic network, we obtain a folding map $T^\dagger \rightarrow F(T)=T^\dagger /\sim _{T^\dagger }$ whose fibres are the equivalence classes of $\sim _{T^\dagger }$.

We now state a result that provides additional insight into unfoldings of networks, and that will also be useful in the last section. It can be regarded as a phylogenetic analogue of path lifting in topology (cf. also Boldi and Vigna 2002, Theorem 13 and Corollary 14).

### **Theorem 4**

Suppose that *T* and $T'$ are pseudo MUL-trees on *X*, that *N* is a phylogenetic network on *X* and that $g:T' \rightarrow N$ is an *X*-morphism. If $f:T \rightarrow N$ is a folding map, then there exists an *X*-morphism $\tilde{g}:T' \rightarrow T$ such that $f \circ \tilde{g} = g$. Moreover, if *g* is a rooted *X*-morphism, then so is $\tilde{g}$, and $\tilde{g}$ is necessarily unique.

### *Proof*

Using a top-down approach, we define $\tilde{g}$ recursively as follows. Since *f* is a folding map, there exists a vertex *u* in $f^{-1}(g(\rho _{T'}))$. We set $\tilde{g}(\rho _{T'})= u$. Now, if the map $\tilde{g}$ has been defined on the parent $v'$ of some $v \in V(T')$ as well as the arcs and vertices on the directed path from $\rho _{T'}$ to $v'$, and $a=(v',v) \in A(T')$, then we define $\tilde{g}(a)=\widetilde{g(a)^{\tilde{g}(v')}}$, and $\tilde{g}(v)$ to be the head of this arc in *T*. It is straight-forward to check that the mapping $\tilde{g}$ that we obtain in this way yields an *X*-morphism with the desired property. Moreover, if *g* is a rooted *X*-morphism, then $\rho _N=g(\rho _{T'})$ and hence $f^{-1}(g(\rho _{T'}))=\{\rho _T\}$. This implies that $\tilde{g}$ is a rooted *X*-morphism, and that $\tilde{g}$ is the only such map. $\square$

As a corollary of this result, we now see that the pseudo MUL-tree $U^*(N)$ can be regarded as a phylogenetic analogue of the *universal total graph* of *N* (at $\rho _N$), a graph theoretical variant of the universal cover of a topological space (cf. Boldi and Vigna 2002, Section 3.1).

### **Corollary 2**

Suppose that $T'$ is a pseudo MUL-tree and *N* is a phylogenetic network, both on *X*, and that $g:T' \rightarrow N$ is a folding map. Then $T'$ is isomorphic to $U^*(N)$.

### *Proof*

Applying Theorem 4 with $T=U^*(N)$ and $f=f^*:U^*(N) \rightarrow N$, it follows that there exists a unique rooted *X*-morphism $\tilde{g}:T' \rightarrow U^*(N)$ with $f \circ \tilde{g} = g$. Since *g* is a folding map, it follows that $\tilde{g}$ is also a folding map, and hence an isomorphism, as required. $\square$

Using again the notation for a guidetree for the operation *F*, we now use this last result to provide an alternative characterisation for stable networks.

### **Corollary 3**

Suppose that *N* is a phylogenetic network. Then *N* is stable if and only if $U^*(N)$ is isomorphic to $[U(N)]^\dagger$.

### *Proof*

Suppose *N* is stable, that is, *N* is isomorphic to *F*(*U*(*N*)). By the comment following Theorem 3, there exists a folding map from the pseudo MUL-tree $[U(N)]^\dagger$ to *F*(*U*(*N*)). As *N* is isomorphic to *F*(*U*(*N*)), there also exists a folding map from $U^*(N)$ to *N*. By Corollary 2, it follows that $U^*(N)$ is isomorphic to $[U(N)]^\dagger$.

Conversely, suppose $U^*(N)$ is isomorphic to $[U(N)]^\dagger$ and write $\sim ^{\dagger }$ rather than $\sim _{[U(N)]^{\dagger }}$. By Theorem 2 we have a folding map $f^*:U^*(N) \rightarrow N$. Hence, by Theorem 3, there exists an equivalence relation $\sim ^*$ on $V(U^*(N))$ such that *N* is isomorphic to $U^*(N)/{\sim ^*}$. Moreover, $u\sim ^* v$ in $V(U^*(N))$ if and only if the pseudo MUL-trees $U^*(N)(u)$ and $U^*(N)(v)$ are isomorphic.

Now, *F*(*U*(*N*)) is isomorphic to $[U(N)]^\dagger /\sim ^\dagger$, where $u' \sim ^\dagger v'$ in $V([U(N)]^\dagger$ if and only if $[U(N)]^\dagger (u')$ is isomorphic to $[U(N)]^\dagger (v')$. Therefore, the two equivalence relations $\sim ^*$ and $\sim ^\dagger$ are equal (up to the isomorphism between $U^*(N)$ and $[U(N)]^\dagger$), and hence *N* is isomorphic to *F*(*U*(*N*)), as required. $\square$

Note that our definition for folding maps can be extended to obtain folding maps between *X*-networks in general. We will not pursue this possibility further here, but it could be of interest to understand categorical properties of such maps (cf. Boldi and Vigna 2002, Section 6).

## Displaying trees in stable networks

**Fig. 5:**
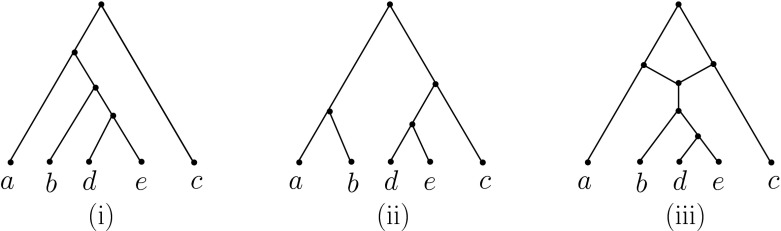
The phylogenetic tree in **(i)** is displayed by the network in **(iii)**, but the tree in **(ii)** is not

Following Iersel et al. (2010), we say that a phylogenetic network *N* on *X* *displays* a phylogenetic tree *T* on *X* if there is a subgraph $N'$ of *N* that is a subdivision of *T* (i. e. $N'$ can be obtained from *T* by replacing arcs (*u*, *v*), $u,v\in V(T')$ by directed paths from *u* to *v*). We illustrate this concept in Fig. 5.

In Kanj et al. (2008) it is shown that it is NP-complete to decide whether or not a given phylogenetic tree is displayed by a given phylogenetic network. On the other hand, in Iersel et al. (2010) it is shown that there are polynomial algorithms for this problem for certain classes of networks e. g. binary tree-child networks. Thus it is of interest to know the complexity of this question for stable phylogenetic networks. We show that the following decision problem is NP-complete.

TreeDisplaying

*Instance:* A binary stable phylogenetic network on *X* and a binary phylogenetic tree on *X*.

*Question:* Is *T* displayed by *N*?

To establish this fact, we show that this problem is NP-complete when restricted to compressed, binary tree-sibling networks and apply Corollary 1. In the proof, we shall use the following operation, which is a modification of an operation with the same name defined in Iersel et al. (2010). Suppose that *N* is a binary phylogenetic network on *X* and that *R* is a binary phylogenetic tree on *X*. Let $\rho _N$ denote the root of *N*, let $\rho _R$ denote the root of *R*, and let $v\in V(N)$. Assume that $x_v,x_v', p_v, q_v,\rho _v$ are pairwise distinct vertices not already contained in *N* and that $x_v$, $x_v'$, $p_v$ and $\rho _v$ are also not contained in *R*. Then the operation HangLeaves( *v* ) adds the vertices $x_v,x_v', p_v, q_v,\rho _v$ to *N* as well as the arcs $(\rho _v, \rho _N)$, $(\rho _v, p_v)$, $(p_v,q_v)$, $(v,q_v)$, $(p_v,x'_v)$ and $(q_v,x_v)$. In addition, it adds the vertices $x_v,x_v',\rho _v, p_v$ to *R* as well as the arcs $(\rho _v,\rho _R)$, $(\rho _v,p_v)$, $(p_v,x_v)$, and $(p_v,x'_v)$.

### **Theorem 5**

TreeDisplaying is NP-complete, even when restricted to the class of binary, compressed tree-sibling networks.

### *Proof*

By Corollary 1, it suffices to restrict attention to the class of binary, compressed tree-sibling networks. Let *T* be a binary phylogenetic tree on *X* and let *N* be a binary phylogenetic network on *X*. We will (in polynomial time) modify *N* to a binary, compressed tree-sibling network $N^*$ on some leaf set $X^*$ that contains *X* and, simultaneously, modify *T* to a binary phylogenetic tree $T^*$ on $X^*$. For $T^*$ and $N^*$ we then show that *T* is displayed by *N* if and only if $T^*$ is displayed by $N^*$. The result then follows as it has been shown in Kanj et al. (2008) that it is NP-complete to decide whether or not a binary phylogenetic tree is displayed by a binary phylogenetic network.

The construction of $N^*$ is in two steps. In the first step, we repeatedly apply the operation HangLeaves to transform *N* into a compressed tree-sibling network on some yet to be specified leaf set $X'$ and *T* to a binary phylogenetic tree on $X'$. To do this we associate to *N* a phylogenetic network $N_1$ in which every reticulation vertex has a unique child and that child is a tree-vertex. This is achieved by carrying out the following operation. For each arc *e* in *N* whose head is a reticulation vertex of *N* we subdivide *e* by a new vertex $v_e$ and then apply HangLeaves to $v_e$. We denote the resulting rooted DAG by $N_1$. Note that $N_1$ is clearly a binary phylogenetic network on *X*, every reticulation vertex of $N_1$ has a unique child, and that child is a tree-vertex. Furthermore, every reticulation vertex of $N_1$ that is also a reticulation vertex of *N* has two siblings in $N_1$ both of which are reticulation vertices.

Next, we follow the proof of Iersel et al. (2010), Theorem 3 and choose for every reticulation vertex *v* of $N_1$ that is also a reticulation vertex in *N* one of its two siblings. Let *s* denote that sibling. Let $p_s$ denote the joint parent of *s* and *v* in $N_1$. Then we subdivide the arc $(p_s,s)$ of $N_1$ by a new vertex $v_s$ and apply HangLeaves to $v_s$. We denote the resulting DAG by $N_2$. Note that $v_s$ is a tree-sibling of *v* in $N_2$, and that $x'_{v_s}$ is a tree-sibling of $q_{v_s}$ in $N_2$. Let $X^*$ denote the union of *X* and all of the leaves added to *N* this way. Then it is easy to check that the resulting DAG $N^*$ is a binary, compressed tree-sibling network on $X^*$. Moreover, the phylogenetic tree $T^*$ constructed in concert with $N^*$ is clearly binary and has leaf set $X^*$.

We now establish our claim that *T* is displayed by *N* if and only if $T^*$ is displayed by $N^*$. To do so, we first show that *T* is displayed by *N* if and only if $T'$ is displayed by $N'$ where $N'$ and $T'$ are a phylogenetic network and a phylogenetic tree on $X'$, respectively, that are the result of a single application of operation HangLeaves, to a vertex *v* of *N*.

Assume first that *T* is displayed by *N*. To see that $T'$ is displayed by $N'$ note first that there exists a subgraph $N''$ of *N* that is a subdivision of *T*. Combined with the fact that the subgraph of $N'$ with vertex set $x_v,x_v', p_v, q_v,\rho _v, \rho _N$ and arc set $(\rho _v, \rho _N)$, $(\rho _v, p_v)$, $(p_v,q_v)$, $(p_v,x'_v)$ and $(q_v,x_v)$ is a subdivision of the subtree of $T''$ of $T'$ whose vertex set is $x_v,x_v',\rho _v, p_v,\rho _T$ and whose arc set is $(\rho _v,\rho _T)$, $(\rho _v,p_v)$, $(p_v,x_v)$, and $(p_v,x'_v)$, it is easy to see that $N''$ gives rise to a subgraph of $N'$ that is a subdivision of $T'$. Thus, $T'$ is displayed by $N'$.

Conversely, assume that $T'$ is displayed by $N'$, that is, there exists a subgraph $N''$ of $N'$ that is a subdivision of $T'$. Clearly, the restriction of $N''$ to $V(N'')-\{x_v,x_v', p_v, q_v,\rho _v\}$ is a subgraph of *N* that is a subdivision of $T'$ restricted to $V(T')-\{x_v,x_v',\rho _v, p_v\}$, that is *T*. Thus, *T* is displayed by *N* which completes the proof of the claim. A repeated application of the last claim implies that *T* is displayed by *N* if and only if $T^*$ is displayed by $N^*$. $\square$

## Weakly displaying trees

Given a phylogenetic tree *T* and a network *N* on *X*, we say that *T* is *weakly displayed* by *N* if it is displayed by *U*(*N*) (that is, there exists a subgraph of *U*(*N*) that is a subdivision of *T*). For example, both of the trees in Fig. 5 are weakly displayed by the phylogenetic network *N*, but the tree in (ii) is not displayed by *N*. As we shall see, this concept is closely related to the problem of reconciling gene trees with species networks. In Sect. 6, we studied the problem of displaying trees in networks, in particular showing that it is NP-complete to decide whether or not a binary phylogenetic tree *T* is displayed by a phylogenetic network *N* even if it is stable. In this section, we show that, in contrast, one can decide in polynomial time whether or not a tree is weakly displayed by any given phylogenetic network.

Before presenting our algorithm, we first derive a characterization for when a tree is weakly displayed by a phylogenetic network in terms of so-called tree reconciliations. Given a phylogenetic network *N* on *X*, let $V_{\mathop {tr}}(N)$ be the set consisting of the tree vertices in *V*(*N*) together with the root on *N*. Following Zhang et al. (2011), a *reconciliation map* between a phylogenetic tree *T* on *X* and a phylogenetic network *N* is a map $r:V(T)\rightarrow V(N)$ such that $r(v)\in V_{\mathop {tr}}(N)$ for all $v\in V(T)$, $r(x)=x$ holds for all $x\in X$, and every arc (*u*, *v*) in *A*(*T*) is associated with a directed path $\mathbf {P}_r(u,v)$ in *N* with initial vertex *r*(*u*) and terminal vertex *r*(*v*).

**Fig. 6:**
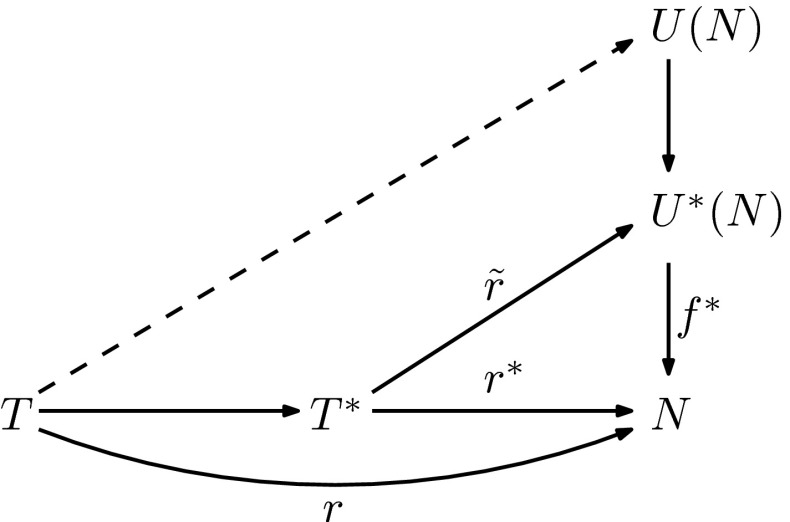
The relationship between the maps described in the proof of Theorem 6

We now give the aforementioned characterization for when a tree is weakly displayed by a phylogenetic network. We call a reconciliation *r* between *T* and *N* *locally separated* if for each pair of vertices $v_1$ and $v_2$ in *T* that have the same parent *v*, both $\mathbf {P}_r(v,v_1)$ and $\mathbf {P}_r(v,v_2)$ contain at least one arc, and the initial arc in $\mathbf {P}_r(v,v_1)$ is distinct from the initial arc in $\mathbf {P}_r(v,v_2)$.

### **Theorem 6**

Suppose that *N* is a phylogenetic network on *X*. Then a phylogenetic tree *T* on *X* is weakly displayed by *N* if and only if there exists a locally separated reconciliation between *T* and *N*.

### *Proof*

We first prove that if there is a locally separated reconciliation *r* between *T* and *N*, then *T* is weakly displayed by *N*. We illustrate the main idea of the proof in Fig. 6—essentially, the map *r* induces an *X*-morphism $r^*$ from a subdivision $T^*$ of *T* into *N*, and so, using Theorem 4, we obtain an *X*-morphism $\tilde{r}$ from $T^*$ to $U^*(N)$, from which we can then deduce that *T* is displayed by *U*(*N*).

More specifically, suppose that *r* is a locally separated reconciliation between *T* and *N*. Since each arc in *T* is associated with a directed path in *N* which contains at least one arc, it follows that *r* induces an *X*-morphism $r^*$ from a subdivision $T^*$ of *T* to *N*. By Theorem 4, let $\tilde{r}$ be an *X*-morphism from $T^*$ to $U^*(N)$ such that $f^*\circ \tilde{r}=r^*$. Since *r* is locally separated, it follows that the map $\tilde{r}$ is injective, and hence $T^*$ is isomorphic to a subgraph of $U^*(N)$.

Now, consider the set $V_0\subseteq V(T^*)$ that is the pre-image of the in-degree one and out-degree one vertices in $U^*(N)$ under $\tilde{r}$. Then, since $\tilde{r}$ is an *X*-morphism, each vertex in $V_0$ has in-degree one and out-degree one. Let $T'$ be the tree obtained from $T^*$ by suppressing all vertices in $V_0$. Then $T'$ is a subdivision of *T*. Since *U*(*N*) does not contain any in-degree one and out-degree one vertices and $U^*(N)$ is a subdivision of *U*(*N*) it follows that $T'$ is isomorphic to a subdivision of $U^*(N)$. Thus, *T* is displayed by *U*(*N*), and so *T* is weakly displayed by *N*, as required.

Conversely, suppose that *T* is weakly displayed by *N*. Then there exists a subdivision $T'$ of *T* such that $T'$ is isomorphic to a subgraph of *U*(*N*). Since $U^*(N)$ is a subdivision of *U*(*N*), there exists a subdivision $T^*$ of $T'$ (and hence also a subdivision of *T*) such that $T^*$ is isomorphic to a subgraph of $U^*(N)$. Denote the *X*-morphism from $T^*$ to $U^*(N)$ induced by this isomorphism by $r^*$ and let $f^*$ be the folding map from $U^*(N)$ to *N* given by Theorem 2. Then the *X*-morphism $f^*\circ r^*$ from $T^*$ to *N* induces a map *r* from *V*(*T*) to *V*(*N*) defined by putting $r(v)=f^*\circ r^*(v)$, for all $v\in V(T)$. Clearly, *r*(*v*) exists because $r^*(v)$ is contained in $V(U^*(N))$ and thus in *V*(*U*(*N*)) as $r^*$ is an *X*-morphism. Moreover, since $f^*$ and $r^*$ are *X*-morphisms it follows that *r*(*v*) is a tree vertex of *N*.

Now, for every arc (*u*, *v*) in *T*, denote the subdivision of (*u*, *v*) in $T^*$ by $P_{u,v}$ (that is, $P_{u,v}$ is the necessarily unique path from *u* to *v* in $T^*$) and let $\mathbf {P}_r(u,v)$ be the image of $P_{u,v}$ under $f^*\circ r^*$, a directed path from *r*(*u*) to *r*(*v*) in *N* which contains at least one arc. Then it follows that *r* is a reconciliation between *T* and *N*. Moreover, to see that *r* is locally separated, consider an arbitrary pair of distinct vertices $v_1$ and $v_2$ in *V*(*T*) that have the same parent *v*. Denote the initial arcs of the two (necessarily distinct) directed paths $P_{v,v_1}$ and $P_{v,v_2}$ in $T^*$ by $a_1$ and $a_2$, respectively. Furthermore, for any arc *a* of *U*(*N*) put $t(a)=t_{U(N)}(a)$ and $h(a)=h_{U(N)}(a)$. Since $r^*$ is induced by an isomorphism between $T^*$ and a certain subgraph of $U^*(N)$, we obtain $r^*(a_1)\not =r^*(a_2)$. Combined with $t(r^*(a_1))=t(r^*(a_2))=v$ and Property (v) in Theorem 3 it follows that $f^*(h(r^*(a_1)))\not =f^*(h(r^*(a_2)))$. Therefore $\mathbf {P}_r(v,v_1)$ and $\mathbf {P}_r(v,v_2)$ contain distinct initial arcs, from which it follows that *r* is locally separated, as required. $\square$

In light of the last result, deciding whether or not a phylogenetic tree is weakly displayed by a phylogenetic network is equivalent to the following decision problem:

Locally separated reconciliation

*Instance:* A phylogenetic network *N* on *X* and a binary phylogenetic tree *T* on *X*.

*Question:* Does there exist a locally separated reconciliation between *T* and *N*?

We now present a dynamic programming algorithm to solve this problem. Let *N* be a phylogenetic network on *X* and let *T* be a binary phylogenetic tree on *X*. Then for every tree vertex *v* in *N* we denote by *N*(*v*) the phylogenetic network obtained from by *N* by first restricting *N* to *v* and all the vertices of *N* below *v* and then suppressing any resulting vertices with in-degree one and out-degree one. In addition, we define a function $\tau : V(T)\times V(N) \rightarrow \{0,1\}$ as follows. If *v* is not a leaf in *V*(*T*), then we set $\tau (v,u)=1$ if and only if there exists some $u'\in V_{tr}(N)$ such that (i) $u'=u$ or $u'$ is below *u* in *N*, and (ii) there exists a locally separated reconciliation between *T*(*v*) and $N({u'})$. If *v* is a leaf with label *x*, then we set $\tau (v,u)=1$ if and only if *u* is a leaf in *N* labeled with *x* or *x* is a leaf in *N* below *u*. We remark that $\tau (v,u)=1$ implies that $\tau (v,u^*)=1$ holds for all $u^*$ such that *u* is below $u^*$ in *N*.

By definition, there exists a locally separated reconciliation between *T* and *N* if and only if $\tau (\rho _T,\rho _N)=1$. In order to compute the value of $\tau (\rho _T,\rho _N)$, we will use the following result concerning the function $\tau$.

### **Proposition 2**

Let *T* be a binary phylogenetic tree on *X*, and *N* a phylogenetic network on *X*. Suppose that *v* is an interior vertex in *T* with two children $v_1$ and $v_2$ and $u\in V(N)$. Then $\tau (v,u)=1$ if and only if *u* is an interior vertex in *N* with $\tau (v,u')=1$ for a child $u'$ of *u*, or there exist two distinct children $u_1, u_2$ of *u* in *N* such that $\tau (v_1,u_1)=1$ and $\tau (v_2,u_2)=1$.

### *Proof*

We begin by establishing the ‘if’ direction. Note that if $\tau (v,u')=1$ holds for a child $u'$ of *u* then, by the previous remark, $\tau (v,u)=1$ follows. Therefore we may assume that *u* is an interior vertex in *N* with two children $u_1\not =u_2$ in *N* such that $\tau (v_1,u_1)=1$ and $\tau (v_2,u_2)=1$. This implies that there exist two (not necessarily distinct) vertices $u'_1$ and $u'_2$ in *N* such that for $i=1,2$, there exists a locally separated reconciliation $f_i$ between $T({v_i})$ and $N({u'_i})$. Fix a directed path $P_i$ in *N* obtained by combining the arc $(u,u_i)$ and an arbitrary path from $u_i$ to $u'_i$. Since $u_1\not =u_2$ the paths $P_1$ and $P_2$ both contain at least one arc and their respective first arcs are distinct.

Now consider the map $f:V(T(v))\rightarrow V(N(u))$ defined, for all $v'\in V(T(v))$, by $f(v')=u$ if $v'=v$, $f(v')=f_1(v')$ if $v'$ is contained in $T(v_1)$, and $f(v')=f_2(v')$ otherwise. Since $v_1$ and $v_2$ are the two children of *v* and $\mathbf {P}_f(v,v_i)=P_i$ holds for $i=1,2$ and $\mathbf {P}_f(v',v'')=\mathbf {P}_{f_i}(v',v'')$ holds for each arc $(v',v'')$ in $T(v_i)$ it follows that *f* is a reconciliation between *T*(*v*) and *N*(*u*). Combined with the fact that $f_1$ and $f_2$ are locally separated, it follows that *f* is also locally separated. Hence, $\tau (v,u)=1$, as required.

Conversely, suppose that $\tau (v,u)=1$ for *v* an interior vertex in *T* and $u \in V(T)$. We may further assume that $\tau (v,u')=0$ for each child $u'$ of *u* as otherwise the proposition clearly follows. Under this assumption, it follows that there exists a locally separated reconciliation *f* between *T*(*v*) and *N*(*u*) with $f(v)=u$.

Now, let $u'_i=f(v_i)$ for $i=1,2$ (where $u'_1$ is not necessarily distinct from $u'_2$). Since $v_i$ is a child of *v* and *f* is a locally separated reconciliation, it follows that $u'_i$ is below *u* and that $\tau (v_i,u'_i)=1$. Considering the two directed paths $\mathbf {P}_f(v,v_i)$ which have the same starting vertex *v* but distinct initial arcs, it follows that there exist two distinct children $u_1$ and $u_2$ of *u* such that $u'_i$ is contained in $N(u_i)$ for $i=1,2$. Together with $\tau (v_i,u'_i)=1$, this implies $\tau (v_i,u_i)=1$, as required. $\square$

Proposition 2 forms the basis of a dynamic programming algorithm for computing $\tau (\rho _T,\rho _N)$ in polynomial time, which we now briefly describe.

Let $m=|V(T)|$, $n=|V(N)|$, and let *k* be the maximum number of children that any vertex in *N* may have. Note first that a topological ordering $\{v_1,\dots ,v_m\}$ of *V*(*T*) (that is, a linear ordering of *V*(*T*) such that $v_i$ is below $v_j$ in *T* implies $j>i$), can be computed in *O*(*m*) time. Similarly, we can compute a topological ordering $\{u_1,\dots ,u_n\}$ of *V*(*N*) in *O*(*kn*) time. Now, noting that $v_m=\rho _T$ and $u_n=\rho _N$, consider the $m\times n$ matrix whose (*i*, *j*)th entry is $\tau (v_i,u_j)$. Then by Proposition 2, it takes *O*(*mnk*) time to fill this matrix and, therefore, to compute $\tau (\rho _T,\rho _N)$. Since a binary phylogenetic tree *T* on *X* has $2|X|-1$ vertices (Semple and Steel 2003, Proposition 1.2.3), we summarize this last discussion in the following corollary.

### **Corollary 4**

Suppose that *T* is a binary phylogenetic tree on *X*, and that *N* is a phylogenetic network on *X*. Then, using a dynamic programming algorithm, it can be decided in

$$\begin{aligned} O(|X| \cdot |V(N)| \cdot \max _{v \in V(N)}|ch(v)|) \end{aligned}$$

time whether or not *T* is weakly displayed by *N*.

## Footnotes

1 A folding map is analogous to an “opfibration” for digraphs (cf. Boldi and Vigna 2002, Definition 4).
